# Antigens of *Mycobacterium tuberculosis* Stimulate CXCR6+ Natural Killer Cells

**DOI:** 10.3389/fimmu.2020.582414

**Published:** 2020-09-28

**Authors:** José Alberto Choreño-Parra, Luis Armando Jiménez-Álvarez, Marcela Muñoz-Torrico, Gustavo Ramírez-Martínez, Luis Antonio Jiménez-Zamudio, Citlaltepetl Salinas-Lara, Ethel Awilda García-Latorre, Joaquín Zúñiga

**Affiliations:** ^1^Escuela Nacional de Ciencias Biológicas, Instituto Politécnico Nacional, Mexico City, Mexico; ^2^Laboratory of Immunobiology and Genetics, Instituto Nacional de Enfermedades Respiratorias “Ismael Cosío Villegas”, Mexico City, Mexico; ^3^Tuberculosis Clinic, Instituto Nacional de Enfermedades Respiratorias “Ismael Cosío Villegas”, Mexico City, Mexico; ^4^Department of Neuropathology, Instituto Nacional de Neurología y Neurocirugía “Manuel Velasco Suárez”, Mexico City, Mexico; ^5^Tecnologico de Monterrey, Escuela de Medicina y Ciencias de la Salud, Mexico City, Mexico

**Keywords:** tuberculosis, *Mycobacterium tuberculosis*, natural killer cells, CXCR6, innate immunity

## Abstract

Natural killer (NK) cells participate in immunity against several pathogens by exerting cytotoxic and cytokine-production activities. Some NK cell subsets also mediate recall responses that resemble memory of adaptive lymphocytes against antigenic and non-antigenic stimuli. The C-X-C motif chemokine receptor 6 (CXCR6) is crucial for the development and maintenance of memory-like responses in murine NK cells. In humans, several subsets of tissue-resident and circulating NK cells with different functional properties express CXCR6. However, the role of CXCR6+ NK cells in immunity against relevant human pathogens is unknown. Here, we addressed whether murine and human CXCR6+ NK cells respond to antigens of *Mycobacterium tuberculosis* (Mtb). For this purpose, we evaluated the immunophenotype of hepatic and splenic CXCR6+ NK cells in mice exposed to a cell-wall (CW) extract of Mtb strain H37Rv. Also, we characterized the expression of CXCR6 in peripheral NK cells from active pulmonary tuberculosis (ATB) patients, individuals with latent TB infection (LTBI), and healthy volunteer donors (HD). Furthermore, we evaluated the responses of CXCR6+ NK cells from HD, LTBI, and ATB subjects to the *in vitro* exposure to CW preparations of Mtb H37Rv and Mtb HN878. Our results showed that murine hepatic CXCR6+ NK cells expand *in vivo* after consecutive administrations of Mtb H37Rv CW to mice. Remarkably, pooled hepatic and splenic, but not isolated splenic NK cells from treated mice, enhance their cytokine production capacity after an *in vitro* re-challenge with H37Rv CW. In humans, CXCR6+ NK cells were barely detected in the peripheral blood, although slightly significative increments in the percentage of CXCR6+, CXCR6+CD49a−, CXCR6+CD49a+, and CXCR6+CD69+ NK cells were observed in ATB patients as compared to HD and LTBI individuals. In contrast, the expansion of CXCR6+CD49a− and CXCR6+CD69+ NK cells in response to the *in vitro* stimulation with Mtb H37Rv was higher in LTBI individuals than in ATB patients. Finally, we found that Mtb HN878 CW generates IFN-γ-producing CXCR6+CD49a+ NK cells. Our results demonstrate that antigens of both laboratory-adapted and clinical Mtb strains are stimulating factors for murine and human CXCR6+ NK cells. Future studies evaluating the role of CXCR6+ NK cells during TB are warranted.

## Introduction

Natural killer cells participate in immune responses against viral and bacterial pathogens (1). In some cases, such as the infection of mice with murine cytomegalovirus (MCMV), the function of NK cells is crucial for the development of protective immunity (2). This protective role of NK cells is mediated by their cytotoxic capacity, allowing the elimination of intracellular reservoirs of infection. Furthermore, NK cells produce a wide range of inflammatory cytokines that shape the effector activities of other innate and adaptive immune cells (1).

Recently, novel functional characteristics that resemble immune memory have been described in NK cells (3–5). These memory-like properties are triggered by antigenic and cytokine priming, allowing NK cells to enhance their effector functions during recall responses. The phenotype of NK cells mediating immunological memory against a particular stimulus is, in some cases, very specific. For instance, Ly49H+ NK cells are protective against MCMV infection (3), whereas liver resident CD49a+ NK cells mount recall responses against haptens in mice (6). In humans, CD94/NKG2C+ NK cells expand during human cytomegalovirus (HCMV) infection (7–9). Similarly, the molecules CD45RO and CD27 have been identified as potential markers of memory-like NK cells among individuals infected with *Mycobacterium tuberculosis* (Mtb) (10–12). Despite this, the relevance of other memory-like NK cell subsets in infectious disorders remains undetermined.

The C-X-C motif chemokine receptor 6 (CXCR6) plays a pivotal role in memory-like responses of murine NK cells (13). The expression of this molecule increases in NK cells from *Rag1*-/- mice after the exposure to non-infectious virus-like particles (VLP) containing antigens from influenza A virus, vesicular stomatitis virus (VSV), and human immunodeficiency virus (HIV). In VLP-treated *Rag1*-/- mice, CXCR6 mediates the recruitment of primed NK cells to the liver, where they reside, maintaining their adaptive properties. During secondary antigenic challenges, hepatic CXCR6+ NK cells re-expand and are able to protect *Rag2*-/-*Il2rg*-/- mice from a lethal infection with influenza A virus and VSV upon adoptive transfer (13). These data suggest that the liver is a reservoir of memory-like CXCR6+ NK cells that may play an important role in protective immunity against infections. In humans, different subsets of circulating and hepatic NK cells also express CXCR6 (14–16). However, there is little evidence about the role of human CXCR6+ NK cells in relevant infectious diseases.

In the current study, we evaluated the responses of memory-like CXCR6+ NK cells to the exposure to Mtb antigens in mice. In addition, we characterized the immune phenotype and expression of CXCR6 in circulating NK cells from humans with distinct clinical forms of tuberculosis (TB). Our results demonstrate that murine CXCR6+ NK cells can respond to Mtb antigens both *in vivo* and *in vitro*. Remarkably, the repetitive administration of mycobacterial preparations to mice enhances the capacity of NK cells to produce cytokines after the *in vitro* re-challenge with the same stimulus. Furthermore, the phenotype and responses of human CXCR6+ NK cells differed between individuals with distinct degrees of immunity to Mtb. Collectively, our findings demonstrate that antigens of Mtb are stimulating factors for murine and human CXCR6+ NK cells.

## Materials and Methods

### Human Samples

We obtained blood samples from individuals with active pulmonary tuberculosis (ATB) and latent tuberculosis infection (LTBI) that attended the TB clinic of the Instituto Nacional de Enfermedades Respiratorias Ismael Cosío Villegas (INER), in Mexico City. The ATB group included symptomatic patients with positive results in sputum smear microscopy, sputum/bronchoalveolar lavage (BAL) culture, and GeneXpert MTB/RIF test (Cepheid, Sunnyvale, CA, United States). The LTBI cohort included asymptomatic individuals in close-contact with ATB patients that tested positive in the QuantiFERON^®^-TB Gold Plus (QFT^®^-Plus) test (QIAGEN, Hilden, Germany). Individuals with primary or acquired immunosuppression, as well as ATB patients receiving anti-TB drugs during the last week before enrollment, were ineligible. A group of ten healthy volunteer donors (HD) was recruited and served as control. These healthy individuals did not report any relevant comorbidity nor history of contact with TB patients and were examined by two independent physicians, which ruled out symptoms of acute illness. Also, HD were subjected to a complete laboratory workup and screened for LTBI by QuantiFERON^®^-TB Gold Plus.

Clinical and demographic data from study participants were retrieved by direct clinical interview, physical examination, and review of their medical records. All participants or their legal guardians provided written informed consent to participate in the study. Blood samples were processed and stored according to the Mexican Constitution law NOM-012-SSA3-2012, which establishes criteria for the execution of clinical research projects in humans. The current study was reviewed and approved by the Institutional Review Board of the INER (project number B04-15).

### Mice

Male C57BL/6 (B6) mice were bred at the INER animal facility. Experimental mice were used between the ages of 6–8 weeks, in accordance with the Institutional Animal Care and Use guidelines at INER in Mexico City, approved under the protocol B04-15.

### Mtb Exposure in Mice

B6 mice were exposed to a cell wall (CW) extract from Mtb H37Rv administered by subcutaneous injection at a concentration of 1 mg/ml diluted in 100 μl of 1× phosphate-buffered saline (PBS). Some mice received subcutaneous PBS and were considered as controls.

### Mouse NK Cell Isolation

At given time points, spleen and liver were collected from B6 mice, and single-cell suspensions were prepared as follows. Spleens were mechanically homogenized by passage through a 40 μm pore size nylon tissue strainer (Falcon; BD Biosciences, San Jose, CA, United States) using a 3 ml syringe plunger. Spleen cell suspensions were treated with ACK buffer to lyse erythrocytes, washed twice, and counted by Trypan’s blue exclusion method. For hepatic single-cell suspensions preparation, livers were perfused with 1× PBS through the portal vein, placed on Petri dishes with complete Dulbecco’s modified Eagle’s medium (cDMEM) containing glucose, L-glutamine, sodium pyruvate, and sodium bicarbonate, and cut into 1 mm pieces using sterile razor blades. Dissected livers were then homogenized by passage through a 70 μm pore size nylon tissue strainer (Falcon; BD Biosciences, San Jose, CA, United States). Homogenates were re-suspended in 40% Percoll solution (Percoll^TM^, GE Healthcare, Sigma-Aldrich, St. Louis, MO, United States), placed over a layer of 70% Percoll, and centrifuged for 25 min at 850 *g*. Hepatic leukocytes were recovered from the 40%/70% Percoll interface, washed twice with cDMEM, treated with ACK solution, and counted. NK cells were enriched from liver and spleen single-cell suspensions using a commercial kit of magnetic beads (NK Cell Isolation Kit II mouse, Miltenyi Biotec, Germany).

### *In vitro* Stimulations

Isolated mouse liver and spleen NK cells were pooled and plated at a density of 2.5 × 10^6^ cells per ml in cDMEM supplemented with 10% fetal bovine serum (FBS). Pooled liver/spleen NK cells were cultured with 25 μg/ml Mtb H37Rv CW at 37°C, 5% CO_2_. After 48 h of Mtb CW exposure, NK cells were collected for flow cytometry and supernatants stored for analysis of IFN-γ production by ELISA (Mouse IFN gamma ELISA Ready-SET-Go!^®^, Affymetrix eBioscience, San Diego, CA, United States). Spleen NK cells were cultured alone in the same conditions and served as controls.

Human peripheral blood mononuclear cells (PBMCs) were isolated by centrifugation gradient using Ficoll-Paque PLUS (GE Healthcare-Life Sciences, Bensalem, PA, United States). PBMCs from HD, LTBI, and ATB individuals were plated at a density of 2.5 × 10^6^ cells per ml in complete Roswell Park Memorial Institute (RPMI-1640) medium supplemented with 2 mM L-Glutamine and 10% FBS, and cultured with 25 μg/ml Mtb H37Rv CW at 37°C, 5% CO_2_, during 48 h. In addition, PBMCs isolated from buffy coats of six HD obtained from the blood bank of INER were stimulated with 25 μg/ml of an Mtb HN878 CW preparation as described above. The H37Rv and HN878 CW preparations were gently provided by Dr. Shabaana A. Khader, from the Department of Molecular Microbiology, Washington University School of Medicine in St. Louis, MO, United States.

### Flow Cytometry

Mouse liver and spleen cell suspensions, and pooled liver/spleen murine NK cells were incubated with fluorochrome-labeled anti-mouse CD3, CD14, NK1.1, and CXCR6 antibodies. Freshly isolated human PBMCs were stained with two different panels of flow cytometry anti-human antibodies: (A) CD3, C14, CD19, CD56, CXCR6, CD49a, CD69, and IFN-γ; (B) CD3, CD14, CD56, and CD16 (Supplementary Table 1). For intracellular staining, cells were fixed and permeabilized using the BD Cytofix/Cytoperm^TM^ kit (BD Biosciences, United States). Cells were acquired using a BD FACS^TM^ Aria II cytometer (BD Biosciences, United States) and gated based on their forward and side scatter characteristics, as well as on fluorescence minus one (FMO) controls for each specific marker using the FACSDiva software. Mouse NK cells were defined as CD3−, CD14−, and NK1.1+, whereas human NK cells were defined as CD3−, CD14/CD19−, and CD56+. The frequency of specific cell types was calculated using Flow Jo (Flow Jo, LLC, Ashland, OR, United States).

### Statistical Analyses

Descriptive statistics were used to characterize the study population clinically. Statistical analyses were performed using GraphPad Prism 8.4.2 (La Jolla, CA, United States). Specific tests are mentioned in figure and table legends. Values of *p* ≤ 0.05 were considered as significant: ^∗^*p* ≤ 0.05, ^∗∗^*p* ≤ 0.01, ^∗∗∗^*p* ≤ 0.001, and ^****^p ≤ 0.0001.

## Results

### Murine CXCR6+ NK Cells Respond to Mtb Antigens

**FIGURE 1:**
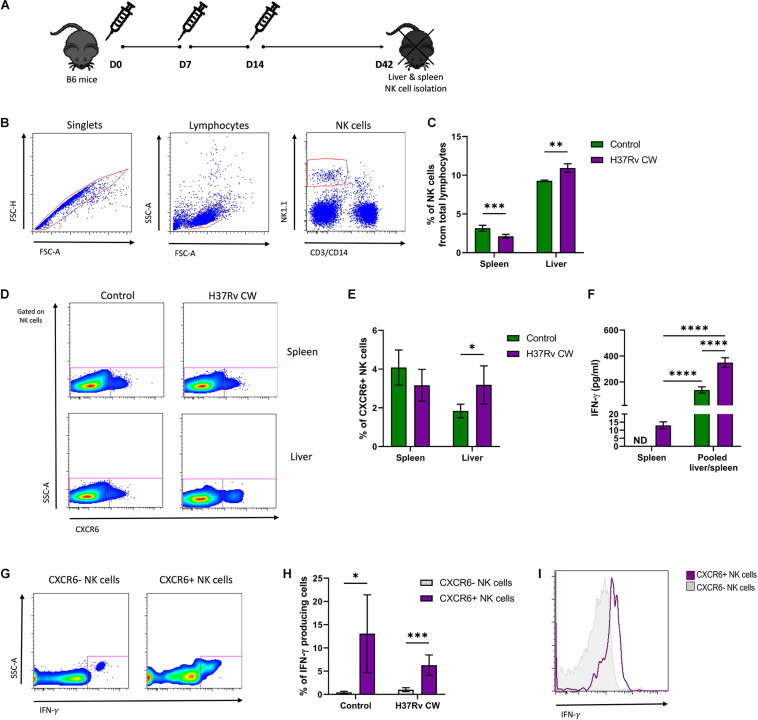
Murine CXCR6+ NK cells respond to Mtb antigens. **(A)** B6 mice were treated with an Mtb H37Rv cell wall (CW) extract administered by subcutaneous injections at days 0, 7, and 14. Another group of mice received 1× phosphate-buffered saline (PBS) and were considered as controls. Thirty days after the last administration, the spleens, and livers from both groups of animals were harvested (*n* = 5 per group). **(B,C)** Percentage of NK cells in total leukocytes from spleen and liver were determined by flow cytometry. **(D,E)** Percentage of splenic and hepatic CXCR6+ NK cells from Mtb H37Rv CW-treated mice and controls were also determined. **(F)** Splenic and hepatic NK cells were isolated from B6 mice treated with the Mtb H37Rv CW and from control animals using antibodies coupled to magnetic beads. Pooled splenic and hepatic NK cells were stimulated *in vitro* with the CW preparation of Mtb H37Rv. After 48 h, levels of IFN-γ in supernatants were quantified by ELISA. The results were compared with the levels of IFN-γ determined in the supernatants of splenic NK cells from treated and control animals that were cultured alone. **(G–I)** Expression of IFN-γ in CXCR6+ NK cells was also determined by flow cytometry. Differences between groups were analyzed using the unpaired Student *t*-test at each tissue and experimental condition. The data shown represent mean (±SE) values from two independent experiments per experimental condition. *p ≤ 0.05, **p ≤ 0.01, ***p ≤ 0.001, and ****p ≤ 0.0001. ND, not detectable.

The chemokine receptor CXCR6+ is a marker of murine memory-like NK cells that mediate recall responses against viruses and haptens (6, 13). To address whether these cells also respond to Mtb, B6 mice were exposed to a CW extract from Mtb H37Rv administered by consecutive subcutaneous injections, as shown in Figure 1A. Four weeks after the last administration, NK cells were quantified in liver, and spleen cell suspensions by flow cytometry (Figure 1B). As compared to naive B6 mice, a significant reduction in the number of total NK cells was observed in the spleen of animals treated with Mtb H37Rv CW. Conversely, mice treated with the mycobacterial CW preparation exhibited an increase in the total amount of hepatic NK cells with respect to the number of NK cells in the liver of naïve B6 mice (Figure 1C). Notably, the proportion of hepatic CXCR6+ NK cells augmented after the *in vivo* exposure to Mtb H37Rv CW in treated animals with respect to naïve B6 mice (Figures 1D,E). These data suggest that the systemic administration of Mtb antigens promotes the expansion of murine CXCR6+ NK cells in the liver.

To address functional changes induced by the *in vivo* exposure to Mtb H37Rv antigens in murine NK cells, we enriched splenic and hepatic NK cells from mice treated with the CW preparation, as well as from naïve animals. However, the amount of hepatic NK cells obtained from each mouse was not enough for *in vitro* assays. Thus, pooled splenic and hepatic NK cells were incubated with the same mycobacterial preparation. After such stimulation, we measured levels of IFN-γ in the supernatants. The results were compared with levels of IFN-γ in supernatants from splenic NK cells isolated from control and treated mice and cultured alone. Interestingly, we found that pooled splenic and hepatic NK cells from mice receiving injections of Mtb H37Rv CW produced higher amounts of IFN-γ after the *in vitro* exposure to the same antigens, as compared to NK cells from naïve mice (Figure 1F). This effect was not observed in splenic NK cells from both groups of animals cultured alone. In fact, the levels of IFN-γ in the supernatants from splenic NK cells were lower than in supernatants from pooled splenic and hepatic NK cells (Figure 1F). This finding suggests that hepatic, but not splenic NK cells, improve their functional capacity after consecutive encounters with Mtb antigens. Notably, we found that a higher proportion of CXCR6+ NK cells participate in the production of such cytokine and express more IFN-γ in response to the CW extract with respect to their CXCR6− counterpart in both treated and control animals (Figures 1G–I). Together, these findings indicate that murine CXCR6+ NK cells actively respond to Mtb antigens.

### CXCR6+ NK Cells in Humans With Pulmonary TB

**FIGURE 2:**
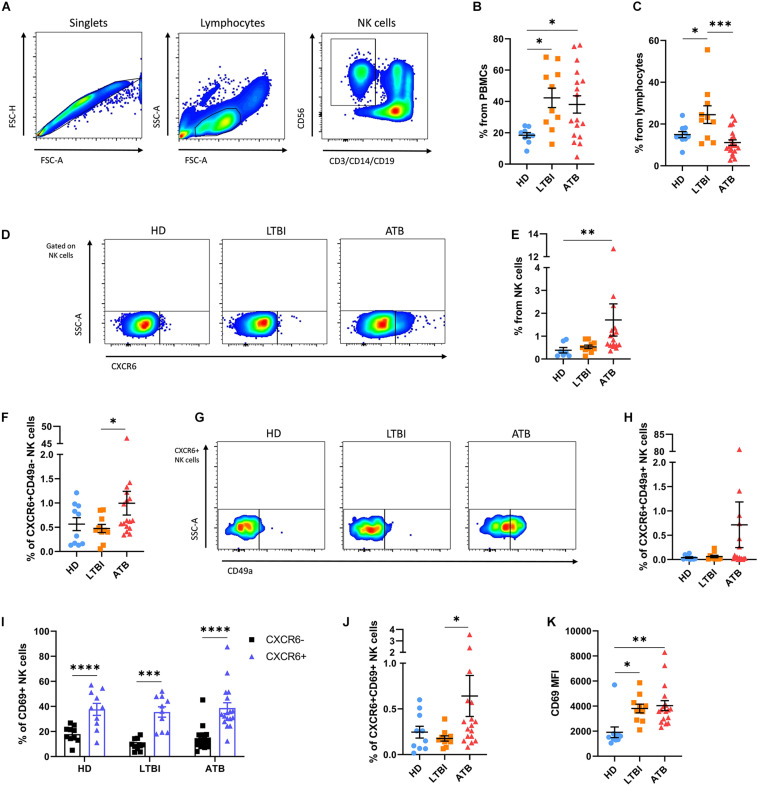
Expression of CXCR6 in NK cells from humans with TB. The immune phenotype of circulating NK cells was analyzed in peripheral blood mononuclear cell (PBMC) samples from healthy donors (HD, *n* = 10), individuals with latent TB infection (LTBI, *n* = 10), and patients with active pulmonary TB (ATB, *n* = 17) by flow cytometry. **(A)** Gating strategy used for the analysis of the immune phenotype of human NK cells. **(B)** Percentage of lymphocytes from total PBMCs. **(C)** Percentage of NK cells from total lymphocytes. **(D,E)** The percentage of CXCR6+ NK cells from total lymphocytes was compared between groups. We also determined the percentage of **(F)** CXCR6+CD49a– NK cells and **(G,H)** double-positive CXCR6+CD49+ NK cells in all participant groups. **(I)** The proportion of cells expressing CD69 were compared between CXCR6+ and CXCR6– NK cells from each group. **(J,K)** Also, the percentage of CXCR6+CD69+ NK cells and mean fluorescence intensity (MFI) values for CD69 in CXCR6+ NK cells were determined in HD, LTBI, and ATB individuals. Differences between groups were analyzed using the one-way ANOVA test and the *post hoc* Tukey’s for multiple comparisons test. Comparisons between cells from the same group were analyzed with the Student *t*-test and *p* values corrected for multiple comparisons using the Holm method. The data shown represent mean (±SE) values. *p ≤ 0.05, **p ≤ 0.01, ***p ≤ 0.001, and ****p ≤ 0.0001.

Using flow cytometry, we characterized the immune profile of circulating NK cells in HD (*n* = 10), individuals with LTBI (*n* = 10), and ATB (*n* = 17) patients. Their main clinical characteristics are summarized in Table 1. The gating strategy used to identify different human NK cell subsets is illustrated in Figure 2A. We observed that LTBI and ATB groups had elevated proportions of circulating lymphocytes as compared to HD (Figure 2B and Supplementary Figures 1A,B). Nonetheless, no differences in the percentage of lymphocytes from total PBMCs were observed between LTBI and ATB patients. Strikingly, NK cells were increased among LTBI individuals as compared to HD and ATB patients (Figure 2C and Supplementary Figure 1C). Also, the proportion of CD56^*bright*^CD16− NK cells was lower in peripheral lymphocytes from ATB patients as compared to HD and LTBI individuals (Supplementary Figure 1D). This coincides with previous reports (17, 18). No differences in peripheral CD56^*dim*^CD16+ NK cells were observed between groups (Supplementary Figure 1E).

**TABLE 1:** Participant characteristics.

**Characteristic**	**HD (*n* = 10)**	**LTBI (*n* = 10)**	**ATB (*n* = 17)**
Median age (range)	35 (24–51)	41 (19–80)	43 (18–64)
Female, *n* (%)	4 (40)	6 (60)	9 (52.94)
BMI, mean (SD)	26.59 (3.39)	28.54 (5.92)	19.92 (3.4)
Diabetes, *n* (%)	0 (0)	0 (0)	8 (47.05)
BCG vaccination	10 (100)	8 (80)	17 (100)
**Drug resistance**			
MDR, *n* (%)	NA	ND	4 (23.52)
Sensitive, *n* (%)	NA	ND	12 (70.58)
Undetermined, *n* (%)	NA	ND	1 (5.88)

*ATB, active pulmonary tuberculosis; BCG, bacillus Calmette-Guerin; HD, healthy volunteer donors; LTBI, latent tuberculosis infection; MDR, multi-drug resistant; NA, not applicable; ND, not determined; SD, standard deviation.*

In humans, CXCR6 marks a subgroup of liver-resident NK cells with a tolerant phenotype that is barely found in peripheral blood under normal conditions (14). As such, we observed little amounts of CXCR6+ NK cells in blood samples from HD and LTBI. Similarly, CXCR6+ NK cells represented a minor proportion of total circulating NK cells in ATB patients, although this latter showed a slight significant increase in the percentage of CXCR6+ NK cells as compared to HD (Figures 2D,E). Indeed, higher percentages of circulating CXCR6+ NK cells were observed in three (∼18%) ATB patients (Figure 2E). CD49a identifies another subset of human intrahepatic NK cells that produce high levels of cytokines upon stimulation (16, 19). Although this subset is almost undetectable in peripheral blood, we found high percentages of NK cells with the phenotype CXCR6-CD49a+ in two HD (20%), three LTBI individuals (30%), and six ATB patients (35%), with no differences between groups, indicating that this population of NK cells are not relevant for TB (Supplementary Figure 2). Conversely, after excluding for CD49a expression, we found that single positive CXCR6+CD49a− NK cells remained increased among ATB patients as compared to the other participant groups (Figure 2F). A third subset of double-positive hepatic CXCR6+CD49a+ NK cells exist and are thought to represent the human counterpart of murine memory-like hepatic CXCR6+ NK cells (15). These cells are immature but have a high capacity to produce inflammatory cytokines like single positive CD49a+ NK cells (15). As for total CXCR6+ NK cells, double-positive CXCR6+CD49a+ NK cells were elevated among a fraction of ATB patients but not in HD and LTBI individuals (Figures 2G,H).

Although the differences described above were not robust, our findings suggest an increased circulation of CXCR6+ NK cells in a minor proportion of ATB patients. Thus, we evaluated the expression of the activation and tissue-homing marker CD69 in CXCR6+ NK cells. Interestingly, a higher percentage of CXCR6+ NK cells expressed CD69 as compared to their CXCR6− counterparts in all participant groups (Figure 2I). However, CXCR6+CD69+ NK cells were increased only in the circulation of ATB patients, but not LTBI individuals and HD (Figure 2J). Finally, CXCR6+ NK cells from LTBI and ATB groups showed a higher relative expression of CD69 as compared to HD (Figure 2K).

### *In vitro* Responses of Human CXCR6+ NK Cells to Mtb Antigens

**FIGURE 3:**
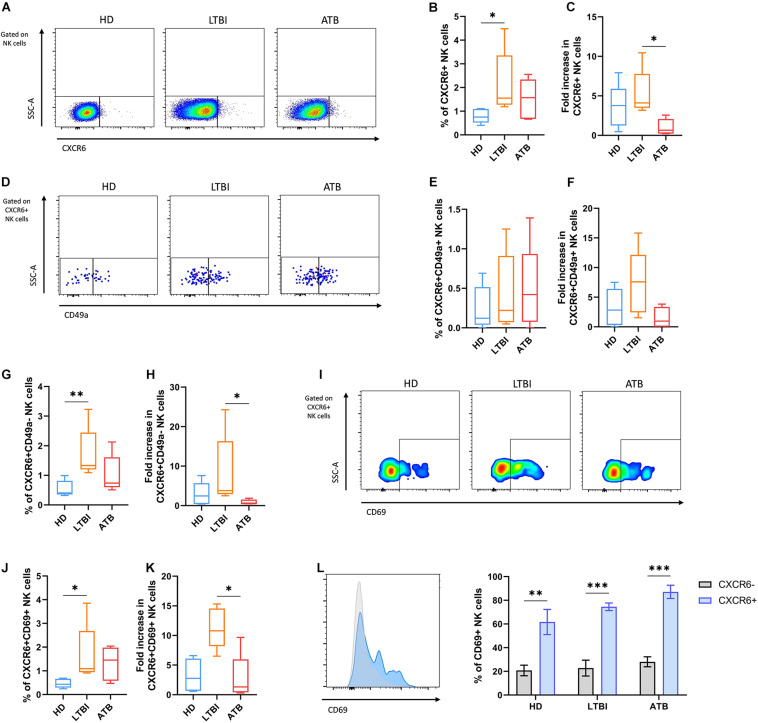
Responses of circulating CXCR6+ NK cells from individuals with TB after the *in vitro* exposure to Mtb antigens. Peripheral blood mononuclear cells (PBMC) from healthy donors (HD), individuals with latent TB infection (LTBI), and patients with active pulmonary TB (ATB) were cultured with a cell wall (CW) extract of Mtb H37Rv for 48 h (*n* = 5 per group). After the *in vitro* stimulation with Mtb antigens, cells were characterized by flow cytometry. Total NK cells were gated as shown in Figure 2A. **(A)** Flow cytometry analysis of CXCR6+ NK cells after the *in vitro* stimulation. **(B)** The percentage and **(C)** fold increase of CXCR6+ NK cells were determined by flow cytometry. **(D,E)** We also determined the percentage and **(F)** fold increase of CXCR6+CD49a+ NK cells. **(G,H)** Comparisons of the percentage and fold increase of CXCR6+CD49a– NK cells between groups were also performed. **(I,J)** Percentage and **(K)** fold increase of CXCR6+CD69+ NK cells determined by flow cytometry. **(L)** The relative expression of CD69 and the proportion of CD69+ cells were compared between CXCR6+ and CXCR6– NK cells at each group. Fold increases were calculated as follows: the percentage of a specific cell subpopulation after the stimulation of PBMCs with Mtb antigens was divided by the percentage of the same cell subset before such stimulation. Differences between groups were analyzed using the Kruskal-Wallis test and the *post hoc* Dunn’s test for multiple comparisons. Comparisons between cells from the same group were analyzed with the Student *t*-test and *p* values corrected for multiple comparisons using the Holm method. The data shown represent mean (±SE) values. *p ≤ 0.05, **p ≤ 0.01, ***p ≤ 0.001.

We evaluated the responses of circulating CXCR6+ NK cells to the *in vitro* exposure with the Mtb H37Rv CW extract using total PBMCs from the last five consecutive enrolled individuals of each participant group. After such antigenic priming, CXCR6+ NK cells represented a higher percentage from total NK cells and showed a more significant fold increase with respect to the baseline percentage only in the LTBI group (Figures 3A–C). Little expression of IFN-γ was observed in stimulated CXCR6+ NK cells, and the percentage of IFN-γ+CXCR6+ NK cells was not different between groups (Supplementary Figure 3). Furthermore, the proportion of NK cells that produce such cytokine was similar in the CXCR6+ and CXCR6− subsets from all participant groups (Supplementary Figure 3).

The antigenic stimulation with the CW preparation of Mtb H37Rv did not induce significant changes in the proportions of double-positive CXCR6+CD49a+ NK cells in the cultures of PBMCs from any group of individuals (Figures 3D–F). Conversely, after excluding for CD49a+ cells, we found that Mtb antigens induced a significant increase in the proportion of single-positive CXCR6+CD49a− NK cells among LTBI individuals, but not HD and ATB patients (Figures 3G,H). The same pattern was observed for CXCR6+CD69+ NK cells, which were elevated only in the PBMCs from LTBI participants after the antigenic priming (Figures 3I–K). Despite this, CXCR6+ NK cells were more responsive to the mycobacterial CW preparation independently of their source as they expressed more CD69 than CXCR6− NK cells in all groups of participants (Figure 3L).

**FIGURE 4:**
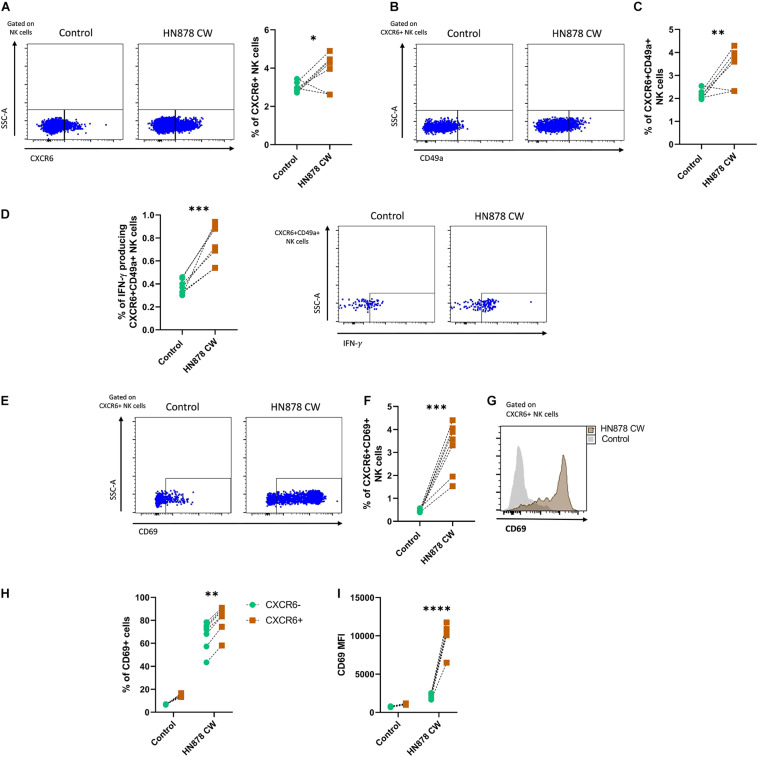
Responses of human CXCR6+ NK cells to the *in vitro* infection with the hypervirulent clinical Mtb strain HN878. Peripheral blood mononuclear cell (PBMC) samples from healthy volunteer donors (HD) were obtained from the blood bank and cultured with a cell wall (CW) extract of the hypervirulent clinical Mtb HN878 for 48 h (*n* = 6). Some PBMCs from the same donors received sterile culture medium and were considered as controls. After the *in vitro* stimulation, cells were characterized by flow cytometry. Total NK cells were gated as shown in Figure 2A. **(A)** The percentage of CXCR6+ NK cells was compared between stimulated and non-stimulated PBMCs. **(B)** Expression of CD49a in CXCR6+ NK cells and **(C)** percentage of CXCR6+CD49a+ NK cells. **(D)** Percentage of IFN-γ producing CXCR6+CD49a+ NK cells. **(E)** Flow cytometry dot-plot showing the expression of CD69 by CXCR6+ NK cells. **(F)** Percentage of CXCR6+CD69+ NK cells and **(G)** relative expression of CD69 in CXCR6+ NK cells. **(H)** The percentage of CD69+ cells and **(I)** mean fluorescence values intensity (MFI) for CD69 were compared between CXCR6+ and CXCR6– NK cells in both stimulated and non-stimulated PBMCs. Differences between groups and between cells from the same group were analyzed with the unpaired Student *t*-test and Mann-Whitney *U* test as appropriate. The data shown represent mean (±SE) values. *p ≤ 0.05, **p ≤ 0.01, ***p ≤ 0.001, and ****p ≤ 0.0001.

Finally, we evaluated whether human CXCR6+ NK cells respond not only to the stimulus with antigens from the lab-adapted Mtb H37Rv strain but also to a CW preparation from a clinically relevant Mtb isolate. Thus, we cultured PBMCs from six healthy individuals with antigens of the prototype W-Beijing lineage Mtb strain, hypervirulent HN878. Our results showed that the Mtb H878 CW extract triggered an increase in the percentage of NK cells expressing CXCR6 (Figure 4A), as well as in the percentage of total- and IFN-γ-producing CXCR6+CD49a+ NK cells (Figures 4B–D). Such a stimulus also resulted in higher expression of CD69 in CXCR6+ NK cells, augmenting the percentage of CXCR6+CD69+ NK cells (Figures 4E–G). This increase in the expression of the tissue-homing marker CD69 was significatively higher in CXCR6+ than in CXCR6− NK cells (Figures 4H,I). Collectively, these data indicate that CW antigens from laboratory-adapted and clinical Mtb strains are stimulating factors for human CXCR6+ NK cells.

## Discussion

Tuberculosis is a leading cause of death worldwide. The incomplete understanding of the mechanisms implicated in protective immunity against Mtb is a barrier to the development of novel and more effective TB vaccines. For many years, the TB vaccine field has relied on the paradigm that IFN-γ-mediated T cell responses are the chief mechanism of protection against Mtb. However, novel insights into the role of innate immune cells in TB are motivating innovative strategies to target several components of the innate immune system and improve Mtb control (20–23). In this regard, NK cells are group 1 innate lymphoid cells (ILC1s) that play a role in the defense against this pathogen (20, 24). Studies in humans have shown that NK cells infiltrate the lung of patients with ATB and localize within tubercle granulomas (25). Also, phenotypical deficiencies of circulating NK cells have been observed among ATB patients with respect to individuals with LTBI. For instance, there is a reduced frequency of CD56^*bright*^ accompanied by decreased expression of the activating receptors NKp30 and NKp46 in peripheral blood NK cells from ATB patients but not LTBI individuals (17, 18). Also, a higher prevalence of inhibitory killer-immunoglobulin like receptors (KIR) has been documented among patients with ATB as compared with resistant individuals (26–29). NK cells also produce a broad range of soluble immune mediators that are known to be crucial for anti-Mtb immunity. These mediators include IFN-γ, TNF-α, IL-17, IL-22, and GM-CSF (2, 30–34). In murine TB models, the production of IL-22 by NK cells is crucial for protective immune responses induced by *Mycobacterium bovis* bacillus Calmette—Guérin (BCG) vaccination (35). Also, in *Rag1*−/− mice infected with Mtb, NK cells can mediate a certain degree of protection against the infection *via* the production of IFN-γ (36). Finally, *in vitro* assays have revealed that NK cells are capable of recognizing several components of Mtb and exert effector activities (37), including cytokine production and cytotoxicity (37–39). Moreover, NK cells can promote the development of protective responses of CD8+ T cells (40), eliminate CD25+ T regulatory cells (41), and even lyse extracellular Mtb (42).

The memory-like properties of NK cells make them an attractive candidate target for TB vaccine development programs (20–23). In this regard, several lymphoid and non-lymphoid tissues are thought to harbor subpopulations of NK cells capable of mediating memory-like immune responses against Mtb (20). For instance, the pleural fluid contains a subgroup of NK cells that express the molecule CD45RO, a marker classically used to identify memory T cells (10, 11). These pleural CD45RO+ NK cells have increased cytotoxic capacity and produce higher levels of inflammatory cytokines in response to IL-12 and BCG as compared to CD45RO- NK cells (10, 11). Similarly, the vaccination of mice with BCG induces an IL-21 dependent expansion of splenic and lymph node NKp46+CD27+KLRG1+ NK cells that mediate protective memory-like responses against Mtb (12). Thus, the memory of NK cells might be an active mechanism of defense during TB (20). However, our knowledge of the mechanisms regulating the function of memory-like NK cells in the context of TB is limited. Moreover, other yet unrecognized subsets of NK cells with specific phenotypes and adoptive properties against Mtb may exist.

In this context, the results of the current study indicate that the repetitive exposure of mice to CW extracts from Mtb H37Rv promote functional changes in murine NK cells, as they improve their cytokine production capacity after consecutive encounters with such mycobacterial components. This coincides with previous reports describing a BCG-induced training of NK cells in humans (43). Nonetheless, our results additionally demonstrate that memory-like NK cell properties induced by mycobacterial components are independent of the persistence of the antigen, as we inoculated mice with CW extracts rather than live Mtb bacilli. Interestingly, the effect described here seems to be restricted to hepatic NK cells from treated mice, as we found enhanced cytokine production only in cultures of pooled hepatic and splenic, but not isolated splenic NK cells re-challenged with Mtb H37Rv CW. This is in agreement with previous studies that revealed that the BCG vaccination does not enhance the capacity of murine splenic NK cells to produce IFN-γ after a re-challenge with BCG *in vitro* (44). Thus, our findings suggest that the liver is an additional reservoir of NK cell subsets with the ability to mount recall responses against Mtb antigens in mice.

One of such hepatic memory-like NK cell subpopulations could be represented by CXCR6+ NK cells, as we found that murine hepatic CXCR6+ NK cells expand *in vivo* after the administration of the Mtb H37Rv CW. This expansion was also independent of the persistence of the antigen. The effects of Mtb antigens are long-lasting, as the phenotypical changes observed in splenic and hepatic NK cells were observed 4 weeks after the last administration of the Mtb H37Rv CW extract. In addition, we demonstrated that CXCR6+ NK cells are capable of responding to direct exposure to Mtb antigens *in vitro*. This subpopulation of murine NK cells has shown memory-like properties in the past (13). Based on our results, these cells also seem to possess an intrinsic higher capacity to produce IFN-γ in response to Mtb antigens as compared to CXCR6− NK cells. These findings may indicate that vaccination with components of Mtb could enhance the functional capacity of CXCR6+ NK cells. However, the effect of the priming of CXCR6+ NK cells on the protective immunity induced by vaccines against Mtb infection was not addressed. Also, changes in murine lung CXCR6+ NK cells in response to the administration of the Mtb H37Rv CW were not evaluated.

To our knowledge, there is no evidence of the role of memory-like CXCR6+ NK cells in respiratory infections. However, the chemotactic axis involving CXCR6 and its ligand CXCL16 might be implicated in defense of the lungs against pathogens. CXCL16 is highly expressed in the sinusoids of the liver and is responsible for the tissue-homing pattern of hepatic CXCR6+ NK cells (45). Interestingly, CXCL16 has also been detected in high concentrations in human BAL specimens. This chemokine is highly expressed by alveolar macrophages, bronchial epithelial cells, airway smooth muscle cells, and lung fibroblasts, and mediates the recruitment of CXCR6+ T cells to the lungs under inflammatory conditions (46, 47). The expression of CXCR6 is upregulated in T cells after pulmonary infection with *Pneumocystis jirovecii* in mice (48). CD4+ and CD8+ T-cells also increase the expression of CXCR6 after mucosal vaccination with antigens from Mtb (49), as well as during pulmonary Mtb infection in murine models of TB (50). Notably, the expression of this chemokine receptor in lung T lymphocytes is associated with vaccine-induced protective immunity against Mtb (49).

Conversely, the role of CXCR6+ T cells is redundant for TB control in unvaccinated mice infected with Mtb. Indeed, CXCR6−/− naïve mice display a lower bacterial burden in the lungs after Mtb infection, as compared to wild type animals (50). This resistant phenotype is also associated with lower IFN-γ production in the lungs of CXCR6−/− mice with pulmonary TB, suggesting that IFN-γ-producing CXCR6+ T cells play pathogenic roles during Mtb infection. In this context, we observed that several subsets of peripheral NK cells (CXCR6+, CXCR6+ CD49a−, and CXCR6+CD49a+) are present at slightly higher frequencies in the blood of a proportion of patients with ATB as compared to LTBI individuals. This may reflect an active mobilization of these cells to the sites of infection during pulmonary TB. Indeed, CXCR6+ NK cells expressing the tissue-homing marker CD69 were found to be increased in the circulation of ATB patients but not HD and LTBI individuals. CD69 is also a marker of activation in lymphocytes (51). Interestingly, the relative expression of this molecule was also increased in both ATB and LTBI subjects. As such, the higher expression of CD69 might be a reminiscent readout of a previous activation in CXCR6+ NK cells from LTBI individuals, whereas it may reflect an ongoing process of activation and tissue-homing in CXCR6+ NK cells from ATB patients. Importantly, we found that the expansion of CXCR6+ NK cells after the *in vitro* exposure to a CW extract from the laboratory-adapted Mtb H37Rv strain was higher in LTBI individuals as compared to ATB subjects. These responses were explained by the expansion of CXCR6+CD49a− NK cells but not double-positive CXCR6+CD49a+ NK cells in LTBI individuals. The former cells are known to display an immature and tolerant phenotype and possess a reduced ability to produce IFN-γ after the antigenic stimulations (14). Meanwhile, double-positive CXCR6+CD49a+ NK cells produce high quantities of IFN-γ upon stimulation (15).

Together, these data indicate that: (a) despite being slightly increased in the circulation of a minor proportion of ATB patients, the subpopulations of circulating CXCR6+ NK cells are less capable of responding to Mtb antigens *ex vivo* as compared to LTBI individuals. Nonetheless, it is possible that memory-like NK cells with enhanced functions against Mtb could be depleted from the circulation and mobilized to the infected lungs. Thus, future studies should look for the presence of CXCR6+ NK cells in lung biopsy or autopsy specimens from ATB patients. (b) Different types of immune cells expressing CXCR6 and producing IFN-γ may play contrasting roles in immunity against murine and human TB. Based on our results and previous studies (50), it is likely that subsets of memory-like NK cells with tolerant phenotypes and reduced capacity of producing IFN-γ, such as hepatic CXCR6+CD49a− NK cells, may play protective roles against Mtb in humans. Similarly, IFN-γ-producing CXCR6+ T cells might be pathogenic during murine TB (50). Unfortunately, the role of other cytokines produced by murine and human memory-like CXCR6+ NK cells in response to Mtb antigens was not addressed in our study. Furthermore, we did not evaluate the cytotoxic capacity of these cells.

Finally, previous studies showed that priming of peripheral blood NK cells with the cytokines IL-12 and IL-15 induces the upregulation of both CD49a and CXCR6 (15). CXCR6+CD49a+ NK cells generated *in vitro* after cytokine priming display phenotypic and functional features similar to liver-resident CD49a+ NK cells, including enhanced IFN-γ expression (15). In line with these findings, we demonstrated that the *in vitro* exposure to clinical strains of Mtb, such as the hypervirulent HN878, induces an increase in the expression of CXCR6 and CD49a in circulating NK cells from healthy volunteer individuals. This stimulation can generate double-positive CXCR6+CD49a+ NK cells with the capacity to produce IFN-γ. Moreover, the CW of Mtb HN878 induces the expression of the activation and tissue-homing marker CD69 in CXCR6+ NK cells. These data add to the evidence about the capacity of different antigenic and non-antigenic stimuli to activate peripheral NK cells, which then acquire tissue-resident properties (15).

The current study has several limitations that must be considered when interpreting our findings. First, the differences in the immunophenotype of human CXCR6+ NK cells between participant groups were not robust. Thus, the protective or pathogenic role of different subsets of CXCR6+ NK cells needs to be elucidated in future studies. Despite this, we found that the responses against the stimulation with Mtb H37Rv CW differed in LTBI and ATB patients. Thus, the main contribution of our manuscript is that we demonstrated that antigens of Mtb stimulate both murine and human CXCR6+ NK cells. Second, even though we found that murine and human CXCR6+ NK cells respond to Mtb antigens, we did not evaluate their mechanisms of activation. In this regard, it is possible that NK cells could have directly recognized Mtb antigens since previous studies have demonstrated the direct activation of NK cells triggered by the toll-like receptor 2 (TLR-2) recognition of CW components from *Mycobacterium bovis* BCG (52). Our results also support this hypothesis, as at least in the case of murine NK cells, we excluded other immune cells from our NK cell cultures using antibodies coupled to magnetic beads. Another possibility is that the responses induced by Mtb antigens in CXCR6+ NK cells could have resulted from the effect of cytokines produced by other immune cells. Indeed, it has been shown that the function of CD4+ T cells is crucial to sustaining the effector activities of NK cells during Mtb infection (53). Thus, T cell-derived cytokines might prime NK cells to respond to Mtb antigens, and even promote the development of cytokine-induced memory-like NK cells in a similar manner to the bystander mechanisms of induction of non-specific memory CD8+ T cells (54). This might apply to the *in vivo* expansion of CXCR6+ NK cells that we observed in mice treated with the Mtb H37Rv CW extract, and to the *in vitro* activation of human CXCR6+ NK cells with the same stimulus, which were cultured as total PBMCs rather than isolated NK cells.

Although our results suggest that hepatic NK cells might develop memory responses against Mtb in mice, we were unable to isolate a sufficient amount of such cells to directly evaluate their functions instead of using pools of splenic and hepatic NK cells. Also, our observations should have been confirmed in RAG1 deficient mice to exclude the interference of T cell memory responses. Despite this, our study constitutes the first evaluation of the capacity of murine and human CXCR6+ NK cells to respond to Mtb antigens and should motivate future investigations to confirm a possible role for these cells in TB.

## Data Availability Statement

The raw data supporting the conclusions of this article will be made available by the authors, without undue reservation.

## Ethics Statement

The studies involving human participants and animal study were reviewed and approved by Institutional Review Board of the Instituto Nacional de Enfermedades Respiratorias “Ismael Cosio Villegas,” Mexico City, Mexico. The patients/participants provided their written informed consent to participate in this study.

## Author Contributions

JC-P participated in the conception of the study, collected clinical data and biological samples from human participants, performed the experiments in mice, and wrote the manuscript. LJ-Á and GR-M participated in experiments with mice and performed flow cytometry. MM-T and CS-L collected clinical and biological samples from human participants and participated in the discussion of the manuscript. LJ-Z, EG-L, and JZ conceived the idea of the study, participated in the analysis of results, and revised the manuscript for intellectual content. All authors contributed to the article and approved the submitted version.

## Conflict of Interest

The authors declare that the research was conducted in the absence of any commercial or financial relationships that could be construed as a potential conflict of interest.
