# Supplementary material for: Antigens of Mycobacterium tuberculosis Stimulate CXCR6+ Natural Killer Cells
Source: Front Immunol. 2020 Sep 28;11:582414. doi: 10.3389/fimmu.2020.582414 (PMC7549382; doi:10.3389/fimmu.2020.582414)
Supplement: Supplementary file 1 [file Data_Sheet_1.PDF]

Supplementary Tables

Supplementary Table 1. Panels of fluorochrome-labeled anti-mouse and anti-human antibodies for NK cells

Marker	Fluorochrome	Clone	Source
**Mouse**			
CD3	APC	17A2	BioLegend
CD14	APC	Sa14-2	BioLegend
NK1.1	BV421	PK136	BioLegend
CXCR6	PE	SA051D1	BioLegend
IFN-γ	PE/Cy7	XMG1.2	BioLegend
**Human (panel A)**			
CD3	FITC	UCHT1	BioLegend
CD14	FITC	HCD14	BioLegend
CD19	FITC	4G7	BioLegend
CD56	PE	HCD56	BioLegend
CD49a	APC	TS2/7	BioLegend
CXCR6	BV605	13B 1E5	BD Biosciences
CD69	BV421	FN50	BioLegend
IFN-γ	PE/Cy7	4S.B3	Bio Legend
**Human (panel B)**			
CD3	BV510	OKT3	Bio Legend
CD14	BV510	M5E2	Bio Legend
CD56	PerCP/Cy5.5	5.1H11	Bio Legend
CD16	APC/Cy7	3G8	Bio Legend

Supplementary Figures

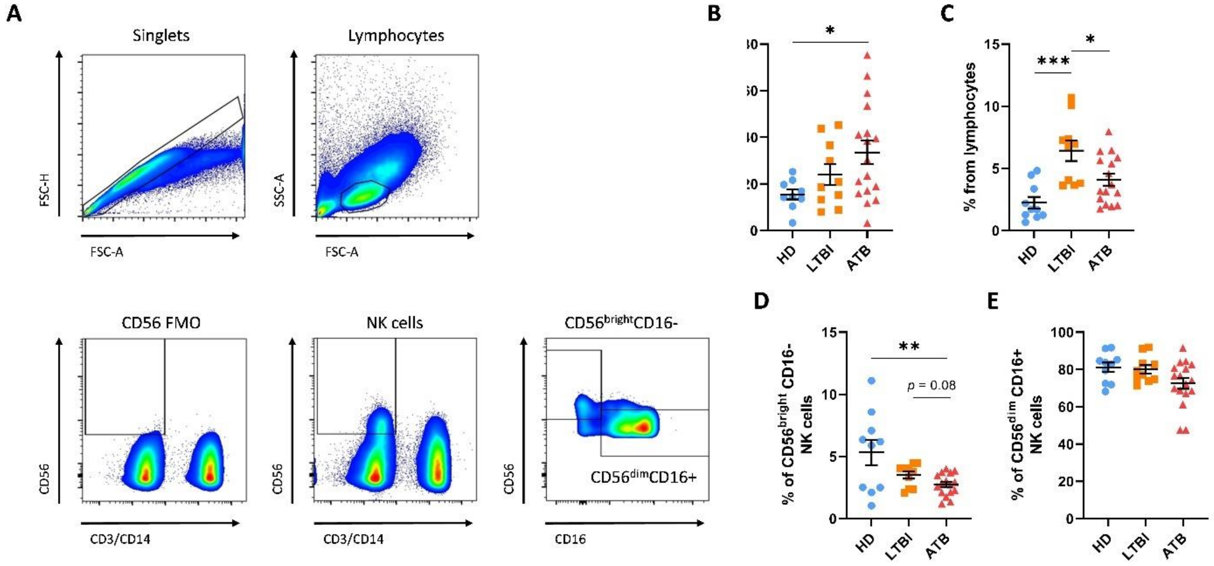

Supplementary Figure 1. Differences in the phenotype of circulating NK cells from individuals with latent TB infection (LTBI) and patients with active TB (ATB) disease. (A) A second panel of fluorochrome-labeled antibodies (human panel B, **Supplementary Table 1**) was parallelly used to evaluate the two major subpopulations of NK cells (CD56^{bright}CD16-, CD56^{dim}CD16+) in peripheral blood mononuclear cells (PBMCs) from healthy volunteer donors (HD, n=10), individuals with latent TB infection (LTBI, n=10), and patients with active pulmonary TB (ATB, n=17). This panel yielded a similar percentage of lymphocytes from total PBMCs **(B)** and percentage of NK cells from total lymphocytes **(C)** than the panel of antibodies (human panel A, **Supplementary Table 1**) used in **Figure 2. (D)** Percentage of CD56^{bright}CD16- NK cells. **(E)** Percentage of CD56^{dim}CD16+ NK cells. Differences between groups were analyzed using the one-way ANOVA test and the post hoc Tukey's for multiple comparisons test. The data shown represent mean (±SE) values. *p≤0.05, **p≤0.01, *** p≤0.001, ****p≤0.0001.

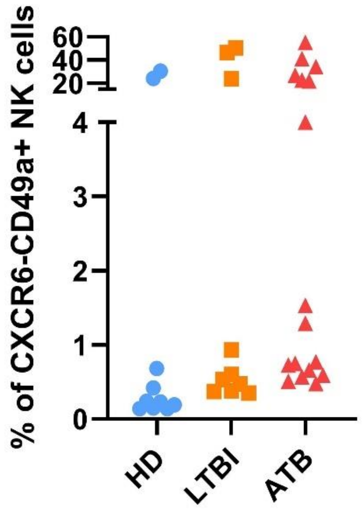

Supplementary figure 2. Percentage of CXCR6-CD49a+ NK cells in healthy donors (HD, n=10), individuals with latent TB infection (LTBI, n=10), and patients with active pulmonary TB (ATB, n=17). Differences between groups were analyzed using the one-way ANOVA test and the post hoc Tukey's for multiple comparisons test. The data shown represent mean (±SE) values.

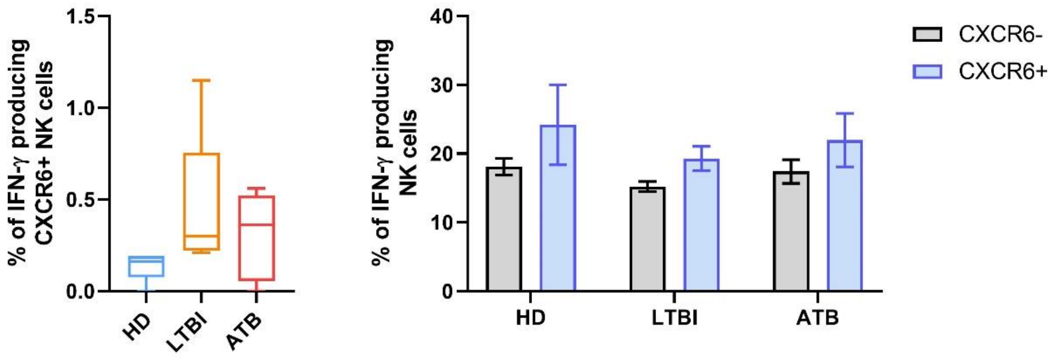

Supplementary figure 3. IFN-γ responses of CXCR6+ NK cells isolated from TB individuals and stimulated with Mtb antigens *in vitro*. Peripheral blood mononuclear cells (PBMCs) from healthy donors (HD), individuals with latent TB infection (LTBI), and patients with active pulmonary TB (ATB) were cultured with a cell wall (CW) extract of Mtb H37Rv for 48 hrs (n=5 per group). After the *in vitro* stimulation with Mtb antigens, we compared the percentage of IFN-γ producing CXCR6+ NK cells between groups. Also, the proportion of CXCR6+ NK cells that produced such cytokine was compared with their respective CXCR6- NK cells counterparts in each group. Differences between groups were analyzed using the Kruskal-Wallis test and the post hoc Dunn's test for multiple comparisons. Comparisons between cells from the same group were analyzed with the Student-T test and *p* values corrected for multiple comparisons using the Holm method. The data shown represent mean (±SE) values. *p≤0.05, **p≤0.01, *** p≤0.001, ****p≤0.0001.
